# Skillful Swimming in Age-Groups Is Determined by Anthropometrics, Biomechanics and Energetics

**DOI:** 10.3389/fphys.2019.00073

**Published:** 2019-02-15

**Authors:** Tiago M. Barbosa, Raul Bartolomeu, Jorge E. Morais, Mário J. Costa

**Affiliations:** ^1^Physical Education and Sport Science Academic Group, Nanyang Technological University, Singapore, Singapore; ^2^Research Centre in Sports Sciences, Health and Human Development (CIDESD), Vila Real, Portugal; ^3^Polytechnic Institute of Bragança, Bragança, Portugal; ^4^Department of Sport Sciences, Exercise and Health, University of Trás-os-Montes and Alto Douro, Vila Real, Portugal; ^5^Polytechnic Institute of Guarda, Guarda, Portugal

**Keywords:** swim stroke, efficiency, hydrodynamics, mechanical power, power input, youth sports

## Abstract

The aim was to compare the anthropometrics, biomechanics and energetics in young swimmers of different competitive levels. Seventy-five boys aged between 11 and 13 years-old with a broad range of performances were ranked based on their personal best time in the men’s 100m freestyle event and then split-up into three tiers (Tier-1, i.e., top-tier, best performers; Tier-2, mid-tier; Tier-3, lower-tier). A set of anthropometric features was measured (height, body mass, arm span and trunk transverse surface area). Stroke kinematics (speed, stroke length, stroke frequency) was assessed by a Speedo-meter. Swim efficiency was then estimated (stroke index, speed fluctuation, Froude efficiency). Hydrodynamics assessment encompassed the estimation of active drag and drag coefficient by velocity perturbation method and a set of dimensionless numbers (Froude, hull speed, Reynolds). Mechanical power (to overcome drag, transfer of kinetic energy to water, external power) and power input were derived. There was a significant variation with moderate effect sizes in all anthropometric features but the trunk transverse surface area. Tier-1 swimmers were taller, heavier and with longer limbs than remaining counterparts. There were also significant variations in the stroke kinematics with moderate-large effect sizes. Tier-1 swimmers showed higher stroke frequency, stroke length, speed, stroke index and propelling efficiency but lower speed fluctuations. Reynold number, Froude number and hull speed were significantly higher in Tier-1 swimmers, denoting large effect sizes. The mechanical power and power input delivered were significantly higher in tier-1 swimmers, showing moderate effect sizes. As a conclusion, it was noted significant variations, with moderate-large effect sizes, among the three tiers, for the vast majority of the selected variables. The better performances by tier-1 swimmers were related to their anthropometrics, biomechanics and energetics.

## Introduction

In endurance sports, notably time-based events, the athlete aims to travel a given distance as quick as possible. In competitive swimming, the speed depends on energetics and biomechanics:

(1) $$\;v=\frac{{\dot{E}}_{tot}}{C}$$

Where *v* is swim velocity, *Ė_tot_* is the total power input (also known as energy expenditure) and *C* the energy cost of swimming. *Ė_tot_* is the sum of the contributions by aerobic and anaerobic (lactic and alactic) energy pathways:

(2) $${\dot{E}}_{tot}={\dot{E}}_{aer}+{\dot{E}}_{anaer-la}+{\dot{E}}_{ATP-PCr}$$

Where *Ė_tot_* is the total power input, *Ė_aer_* the energy contribution by aerobic system, *Ė_anaer-la_* by the anaerobic lactic system, and *Ė_ATP-PCr_* by the anaerobic alactic system. Aerobic energy release is straightforward to be measured because there is a relationship between oxygen uptake measured by breathing (respiratory parameters) and the whole-body aerobic production of ATP; while, methods to measure anaerobic sources are less reliable as anaerobic ATP production takes place at intracellular level, with little reliance on central processes (Gastin, 2001). Standard procedures to monitor anaerobic energetics include assessment of blood lactate, oxygen debt, power output on ergometric test and analytical procedures. Most reliable procedures so far are highly invasive, such as needle biopsy that enables direct measurement of ATP, PCr, pyruvate or lactate concentrations. Future developments in whole-body magnetic resonance could be a true breakthrough in this field. Meanwhile out of all non-invasive options available, oxygen debt seems to be the one providing better insights (Gastin, 2001). Gastin (2001) reported a 45% contribution by aerobic pathways in maximal bouts that take 1 min. Capelli et al. (1998) noted a contribution of 30–35% by aerobic pathways swimming 91 m (100 yards). Conversely, Ribeiro et al. (2015) reported a contribution of 45–50% in the 100 m. In a longer race, the 200 m event, the aerobic contribution has been reported as 60–65% (Capelli et al., 1998; Figueiredo et al., 2011). In all these researches anaerobic lactic contribution was estimated by blood measures and anaerobic alactic contribution by an analytical model. In single and repeated high intensity tethered or free swimming that takes about 30 s the aerobic contribution assessed by accumulated oxygen demand was 25–30% (Peyrebrune et al., 2014). Computational simulations estimated that aerobic contribution in a 100 m event by a world-class swimmer (delivering a time of 48 s) would be 41% (Rodríguez and Mader, 2011). Altogether, in a swimming event that takes just under 1 min (e.g., 100 m) the aerobic contribution to total energy expenditure is 30–45%, depending on the measuring techniques used. However, only one portion of *Ė_tot_* is used for translation of the body’s center of mass. The denominator in equation 1, the energy cost, depends on subject’s swimming proficiency:

(3) $$C=\;\frac{w_{d}}{\eta_{\text{m}}\cdot \;\eta_{\text{p}}}$$

Where *C* represents the energy cost, *w_d_* the mechanical work to overcome drag, η_m_ the mechanical efficiency (also known as gross or overall efficiency) and η_p_ the propelling efficiency. The numerator in equation 3 is affected by swim speed (*D* = Kv^2^) and anthropometrics, such as the surface area (*K* = 1/2⋅ρSCd). η_m_ is the amount of power input that will be used to produce internal and external power $[\eta_{\text{m}}\;=\;({\dot{w}}_{int}+{\dot{w}}_{ext})/{\dot{E}}_{tot}].$ Therefore, a significant amount of *Ė_tot_* (∼80%) is not used for body’s motion but other physiological functions, such as thermoregulation (Pendergast et al., 2003). Hence, if most energy is used for basic physiological functions, η_m_ is less sensitive to swimmer’s level of proficiency. η_p_ is the amount of total work or power $\left({\dot{w}}_{tot}\;=\;{\dot{w}}_{int}+{\dot{w}}_{ext};\;\;\;where\;\;\;{\dot{w}}_{ext}\;=\;{\dot{w}}_{d}+{\dot{w}}_{k}\right)$ that is used to overcome drag force and displace in water (η_p_ = *ẇ_d_/ẇ_tot_*). If one does not includes the *ẇ_int_* term, then this ratio is coined as Froude efficiency (η_F_). If *ẇ_int_* is negligible in human swimming, both propelling and Froude efficiencies can be used interchangeably (η_p_ = η_F_). The amount of work or power used to translate the body in water (η_p_ or η_F_) is indeed sensitive to swimmer’s level of proficiency. Skilful swimmers are expected to yield larger efficiency. Age-group swimmers have been noted as having η_F_ of about 30% and the best age-group swimmers up to about 45% (Barbosa et al., 2015a). In adult national level swimmers, it is on average 60% (Toussaint, 1990) and, Olympic finalists and medallists over 70% (Huang et al., 2010).

Altogether, in order to deliver better performances, swimmers can either enhance *Ė_tot_* (energetics) and/or *C* (biomechanics). Despite we do have a solid body of knowledge on adult and elite swimmers, it is not so clear what are the performance determinants of young counterparts. In young swimmers, 80% of the performance is explained by both energetics and biomechanics combined (Barbosa et al., 2010). The follow-up question is which factor is more determinant. Structural equation modeling predicted that at these early ages, biomechanics and anthropometrics account to 60% of the performance in the 100 m freestyle (Morais et al., 2012). As such, it should be paramount to design holistic and well-rounded protocol to monitor and classify young swimmers’ anthropometrics, biomechanics and energetics. A few cross-sectional (Barbosa et al., 2014) and longitudinal (Morais et al., 2015; Barbosa et al., 2015b) researches assessed the anthropometrics and biomechanics, but missed to measure or estimate the energetics. So, most efforts have been done to understand the role played by the terms in equation 3.

One can argue that experimental procedures to assess energetics in age-group swimmers may have several challenges. The size of snorkels and masks to measure *Ė_aer_* and estimate *Ė_anaer_* by EPOC are not designed for young swimmers and can constrain significantly their swimming technique. Standard procedures to measure *Ė_anaer-la_* are slightly invasive requiring at least a few drops of blood. Assumptions to estimate *Ė_ATP-CP_* based on the amount of active lean mass and rate of utilization of PCr stores are underpinned by experimental research on adults. Hence, a convenient and feasible alternative might be to estimate *Ė_tot_* deriving it from equations 1 and 3.

The aim was to compare the anthropometrics, biomechanics and energetics in young swimmers of different competitive levels. It was hypothesized that top-tier swimmers would have beneficial anthropometric traits, better biomechanical and energetics responses than low-tier counterparts.

## Materials and Methods

### Participants

Seventy-five young boys between 11 and 13 years-old in Tanner stages 1–2 by self-report were recruited. The sample included swimmers with a broad range of performances. All participants were enrolled in development swimming programs at local clubs or national teams and engaged in competitive swimming for at least 2 years. Participants were ranked based on their personal best time in the men’s 100 m freestyle event and then split-up into three tiers (Tier-1, i.e., top-tier, best performers; Tier-2, mid-tier; Tier-3, lower-tier; personal best: Tier-1 = 1.75 ± 0.07 m/s, Tier-2 = 1.53 ± 0.11 m/s, Tier-3 = 1.38 ± 0.13 m/s) of 25 swimmers each. Tier-1 swimmers were at that time age-group national champions, national record holders and/or enrolled in a talent ID program. Tier-2 swimmers were swimmers racing at national competitions. Tier-3 swimmers raced mostly at local and regional competitions.

Written informed consent was provided by both parents or guardians and the swimmers to be participate in this study. Verbal consent was also provided by coaches. All procedures were in accordance to the Helsinki Declaration regarding Human research. The University Ethics Board approved the research design.

### Anthropometrics

Height (H, in m) was measured by a stadiometer (SECA, 242, Hamburg, Germany) and body mass (BM, in kg) on a weighting scale (Tanita, BC-545, Tokyo, Japan). Both measures were taken standing in the upright position, barefoot, and swimwear. Arm span (AS, in m) was measured with swimmers in the upright position, arms and fingers fully extended in lateral abduction at 90° to torso. The distance between fingertips of the 3rd fingers was measured with an anthropometric tape (RossCraft, Canada) (ICC = 0.99).

The trunk transverse surface area (TTSA) was assessed by a photogrammetric technique (Morais et al., 2011). Swimmers were photographed by a digital camera (Nikon, s9600, Tokyo, Japan) in the transverse plane (downward view). Subjects stood in the upright and streamlined position in swimwear, cap, and goggles. A calibration pole (0.945 m) was aside the swimmers’ shoulders. The TTSA was measured by an area measuring software (Udruler V3.0.1211, AVPSoft, United States) (ICC = 0.98).

### Stroke Kinematics

Swimmers were invited to undergo three all-out trials of 25 m at front-crawl and push-off start (at least 30 min of rest). A Speedo-meter string (Swim Speedo-meter, Swimsportec, Hildesheim, Germany) was attached to the swimmers’ hip (Barbosa et al., 2013). The Speedo-meter was set on the forehead-wall of the swimming pool, about 0.2 m above water surface. An in-house built software (LabVIEW^®^, v. 2010) was used to acquire (*f* = 50 Hz) and display speed-time data over each trial. Data was transferred from the Speedo-meter to interface by a 12-bit resolution acquisition card (USB-6008, National Instruments, Austin, TX, United States). Then, it was imported into a signal processing software (AcqKnowledge v. 3.9.0, Biopac Systems, Santa Barbara, CA, United States). Signal was handled with Butterworth 4th order low-pass filter (cut-off: 5Hz, based on the analysis of the residual error vs. cut-off frequency output).

Mean swim velocity over the trial (between about the 11th and 24th meters mark) was measured. Each time the swimmer began a new stroke cycle the researcher would insert a mark on the speed-time curve being displayed on the screen. These events were then used to calculate the stroke frequency (*SF* = 1/*P*, where *P* is the Period). The *SL* was calculated from the *v* and stroke frequency (SF) collected by the Speedo-meter:

(4) $$SL=\;\frac{v}{SF}$$

Where *SL* (in m) is the stroke length, *v* is the swimming velocity (in m/s) and *SF* is the stroke frequency (in Hz). The *SL* was also normalized to the arm span (SL / AS, dimensionless).

### Swim Efficiency

A set of three parameters was used to assess the swim efficiency. The stroke index, as an overall swimming efficiency estimator (Costill et al., 1985):

(5) SI=SL ⋅v

Where *SI* (in m^2^/s) is the stroke index, *SL* (in m) is the stroke length and *v* (in m/s) is the swimming velocity. Another parameter was the intra-cyclic variation of the horizontal velocity of the hip (Barbosa et al., 2013):

(6) $$dv=\frac{\sqrt{\frac{\sum_{i}{\left(v_{i}-\bar{v}\right)}^{2}\cdot F_{i}}{n}}}{\frac{\sum_{i}v_{i}\cdot F_{i}}{n}}\cdot 100$$

Where *dv* (dimensionless) is the intra-cyclic variation of the horizontal velocity of the hip, *v* the mean swimming velocity, *v_i_* the instant swimming velocity, *F_i_* the acquisition frequency and *n* the number of data points. The *dv* mean value of three consecutive stroke cycles between the 11th and 24th mark was used for further analysis (ICC = 0.99). The last parameter selected was the Froude efficiency (Zamparo, 2006):

(7) $${\text{η}}_{\text{F}}=\left(\frac{v\cdot 0.9}{2\text{π}\cdot SF\cdot l}\right)\cdot \frac{2}{\text{π}}$$

Where η_F_ (dimensionless) is the Froude efficiency, *v* (in m/s) the swimming velocity, *SF* (in Hz) the stroke frequency and *l* (in m) the shoulder to hand average distance. The *l* was measured between the acromion and tip of the third finger, on dry-land, with swimmer simulating a stroke cycle, with a measuring tape (RossCraft, Canada; ICC = 0.99 for absolute agreement).

### Hydrodynamics

The velocity perturbation method (VPM) was selected to estimate the active drag (Kolmogorov and Duplischeva, 1992). Active drag was calculated from the difference between the maximal swimming velocities at front-crawl in two all-out trials (with and without towing a perturbation buoy after push-off start). Swimming velocity was measured after clocking the all-out trials between the 11th and 24th meters of the starting wall with stopwatches (Golfinho Sports MC 822, Aveiro, Portugal) by two expert evaluators (ICC = 0.98) and the mean value was used for further analysis (Marinho et al., 2010). Active drag (*D_a_*) was calculated as (Kolmogorov and Duplischeva, 1992):

(8) $$D_{a}=\frac{D_{b}\cdot v_{b}\cdot v^{2}}{v^{3}-v_{b}^{3}}$$

where *D_a_* (in N) is the active drag at maximal velocity, *D_b_* the resistance of the perturbation buoy provided by the manufacturer and, *v_b_* and *v* (in m/s) are the swimming velocities with and without the perturbation device, respectively. Active Drag coefficient was calculated after re-arranging equation 9:

(9) $$C_{Da}=\frac{2\cdot D_{a}}{\text{ρ}\cdot S\cdot v^{2}}$$

Where *C_Da_* (dimensionless) is the active drag coefficient, ρ the density of the water (being 1000 kg/m^3^), *D_a_* the active drag (in N), *v* the swimming velocity (in m/s) and *S* the swimmer’s projected frontal surface area (or TTSA collected with the photogrammetric technique, in m^2^).

The Froude number is deemed as a good proxy of wave-making drag (Kjendlie and Stallman, 2008):

(10) $$Fr=\frac{v}{\sqrt{g\cdot H}}$$

Where *Fr* (dimensionless) is the Froude number, *v* (in m/s) the swimming velocity, *g* the gravitational acceleration (being 9.81 m/s^2^), and *H* (in m) the swimmer’s height. Hull velocity, i.e., the speed at *Fr* = 0.42 (Vogel, 1994) was also selected:

(11) $$v_{h}=\sqrt{\frac{g\cdot H}{2\cdot \text{ π}}}$$

Where *v_h_* (dimensionless) is the hull velocity, *g* the gravitational acceleration (being 9.81 m/s^2^) and *H* (in m) the height. The Reynolds number was used to assess the water flow status around the swimmer:

(12) $$Re=\;\frac{v\cdot H}{\vartheta }$$

Where *Re* (dimensionless) is the Reynolds number, *v* (in m/s) the swimming velocity, *H* (in m) the height, and *υ* the water kinematic viscosity (being 8.97 × 10^-7^ m^2^/s at 26°C).

### Mechanical Power

It was estimated the *ẇ_ext_, ẇ_d_* and *ẇ_k_* as measures of power output. The *ẇ_d_* was computed as:

(13) $${\dot{w}}_{d}=D_{a}\cdot v$$

Where *ẇ_d_* (in W) is the power to overcome drag force, *D_a_* (in N) the active drag and *v* (in m/s) the swimming velocity. The *ẇ_ext_* was computed having as known variables the *ẇ_d_* and η_F_ (Barbosa et al., 2015a):

(14) $${\dot{w}}_{ext}=\frac{{\dot{w}}_{d}}{{\text{η}}_{\text{F}}}$$

Where *ẇ_ext_* (in W) is the external mechanical power, *ẇ_d_* (in W) the power to overcome drag force and η_F_ (dimensionless) the Froude efficiency. Thereafter, *ẇ_k_* was obtained subtracting *ẇ_d_* to *ẇ_ext_*:

(15) $${\dot{w}}_{k}={\dot{w}}_{ext}-{\dot{w}}_{d}$$

Where *ẇ_k_* is the mechanical power to transfer kinetic energy to water, *ẇ_ext_* the external mechanical power, *ẇ_d_* the power to overcome drag force. The total power input is estimated as (Pendergast et al., 2015):

(16) $$\dot{E}tot\;\;=\;\frac{{\dot{w}}_{d}}{{\text{η}}_{\text{m}}\;\cdot \;{\text{ η}}_{\text{F}}}$$

Where *Ė_tot_* (in W) is the total power input, *ẇ_d_* the power to overcome drag, η_F_ (dimensionless) the Froude efficiency and η_m_ the mechanical efficiency. It was assumed an average η_m_ of 0.2 as reported in experimental studies assessing the full stroke (i.e., arm pull plus flutter kicking) (Pendergast et al., 2003; Zamparo et al., 2005). Experimental studies where participants performed the arm-pull with no kicking and/or researchers did not measure/estimated the *ẇ_int_* noted lower η_m_ (Toussaint et al., 1990; Cappaert et al., 1992). Hence, these lower values might be partly due to incomplete computation of all mechanical factors that determine *ẇ_tot_* (Zamparo and Swaine, 2012).

### Statistical Analyses

Sample power was computed having as inputs an error probability of 0.05, *f* effect size of 0.40 and a power of 0.85 to run ANOVAs 1-way (3 groups). These yielded a total sample size of at least 72 subjects (G^∗^Power, v.3.1.9.2, University of Kiel, Germany). Mean ± one standard deviation and 95% confidence intervals are reported for all dependent variables.

Data variation across tiers was analyzed by ANOVA 1-way, followed-up by Bonferroni *post hoc* test (*p* ≤ 0.05). Partial eta-squared ($\eta_{p}^{2}$) was selected as variance effect size index and deemed to be with: (i) small if $\eta_{p}^{2}$ < 0.01; (ii) medium 0.01 < $\eta_{p}^{2}$ < 0.06 and; (iv) large $\eta_{p}^{2}$ > 0.06. Cohen’s d was also selected but as standardized effect size of mean comparisons: (i) | d|< 0.2 trivial; (ii) 0.2 < | d|≤ 0.5 medium; (iii) | d| > 0.5 large.

## Results

### Anthropometrics

Tier-1 and Tier-2 swimmers were significantly taller, heavier and with longer limbs than Tier-3 swimmers; albeit, there were no differences between Tier-1 and Tier-2 participants (Table 1).

**Table 1:** Comparison of the anthropometric features.

	Descriptive			ANOVA			*Post hoc*		
	Tier-1	Tier-2	Tier-3	F-ratio	P	$\eta_{p}^{2}$	T1vT2	T1vT3	T2vT3
	Mean ± 1SD	Mean ± 1SD	Mean ± 1SD	(2;72)			p	p	p
	(95CI)	(95CI)	(95CI)				(d)	(d)	(d)
BM	56.61 ± 9.06	50.18 ± 6.52	48.75 ± 8.89	6.448	0.01	0.15	0.02	<0.001	1.00
[kg]	(53.32;59.89)	(46.90;53.46)	(45.47;52.03)				(0.82)	(0.88)	(0.18)
H	1.67 ± 0.06	1.62 ± 0.05	1.58 ± 0.08	7.991	0.01	0.18	<0.001	<0.001	0.49
[m]	(1.64;1.70)	(1.58;1.62)	(1.55;1,62)				(0.91)	(1.27)	(0.60)
AS	1.75 ± 0.08	1.68 ± 0.06	1.69 ± 0.09	12.557	<0.001	0.26	<0.001	<0.001	0.54
[m]	(1.72;1.78)	(1.65;1,70)	(1.62;1.68)				(0.99)	(0.71)	(0.13)
TTSA	750 ± 123	708 ± 150	718 ± 143	1.024	0.37	0.03	0.83	0.55	1.00
[cm^2^]	(693;808)	(649;763)	(639;753)				(0.31)	(0.24)	(0.07)


### Stroke Kinematics

As expected, there were variations in speed across all groups, being Tier-1 cohort significantly fastest (Table 2). Tier-1 and Tier-2 swimmers denoted the same long *SL*, with no-significant differences; conversely, Tier-2 and Tier-3 swimmers had the same slow *SF*, also with no-significant differences. Therefore, Tier-1 swimmers performed a longer *SL* and faster *SF* than all other swimmers under analysis. Tier-2 swimmers were able to deliver the same *SL* as Tier-1 but the *SF* was similar to Tier-3 counterparts.

**Table 2:** Comparison of the stroke kinematics.

	Descriptive			ANOVA			*Post hoc*		
	Tier-1	Tier-2	Tier-3	F-ratio	P	$\eta_{p}^{2}$	T1vT2	T1vT3	T2vT3
	Mean ± 1SD	Mean ± 1SD	Mean ± 1SD	(2;72)			p	p	p
	(95CI)	(95CI)	(95CI)				(d)	(d)	(d)
SF	0.93 ± 0.11	0.86 ± 0.09	0.88 ± 0.10	4.633	0.01	0.11	0.08	0.01	1.00
[Hz]	(0.89;0.97)	(0.82;0.90)	(0.81;0.88)				(0.70)	(0.48)	(0.21)
SL	1.65 ± 0.21	1.59 ± 0.15	1.56 ± 0.19	10.372	<0.001	0.22	0.44	<0.001	0.01
[m]	(1.59;1.72)	(1.51;1.65)	(1.37;1.51)				(0.33)	(0.45)	(0.18)
SL/AS	94.49 ± 10.09	94.62 ± 10.18	87.24 ± 8.63	4.775	0.001	0.11	1.00	0.03	0.03
[%]	(90.64;98.35)	(90.77;98.47)	(83.39;91.09)				(0.01)	(0.77)	(0.78)
Speed	1.51 ± 0.06	1.35 ± 0.04	1.19 ± 0.08	139.047	<0.001	0.79	<0.001	<0.001	<0.001
[m/s]	(1.48;1.53)	(1.33;1.38)	(1.17;1.22)				(3.14)	(4.53)	(2.53)


### Swim Efficiency

As far as efficiency is concerned, *SI* was significantly different across all groups and better in Tier-1 swimmers (Table 3). η_F_ yielded mixed results, yet mean values were significantly higher in Tier-1 than Tier-3. The *dv* was significantly higher in low-tier swimmers than the other two groups. Altogether, Tier-3 swimmers were less efficient that their counterparts.

**Table 3:** Comparison of the swim efficiency.

	Descriptive			ANOVA			*Post hoc*		
	Tier-1	Tier-2	Tier-3	F-ratio	P	$\eta_{p}^{2}$	T1vT2	T1vT3	T2vT3
	Mean ± 1SD	Mean ± 1SD	Mean ± 1SD	(2;72)			p	p	p
	(95CI)	(95CI)	(95CI)				(d)	(d)	(d)
*SI*	2.51 ± 0.37	2.14 ± 0.21	1.70 ± 0.27	46.963	<0.001	0.57	<0.001	<0.001	<0.001
[m^2^/s]	(2.39;2.62)	(2.02;2.25)	(1.59;1.82)				(1.23)	(2.50)	(1.82)
η_F_	27.77 ± 6.82	26.53 ± 6.42	23.24 ± 6.14	3.278	0.04	0.08	1.00	0.04	0.23
[%]	(25.19;30.35)	(23.96;29.12)	(20.66;25.82)				(0.19)	(0.70)	(0.53)
*dv* [dimensionless]	0.083 ± 0.023	0.084 ± 0.020	0.104 ± 0.042	5.162	0.01	0.13	1.00	0.02	0.03
	(0.073;0.092)	(0.075;0.092)	(0.087;0.121)				(0.05)	(0.62)	(0.62)


### Hydrodynamics

Comparing the hydrodynamics, parameters that are strongly dependent on speed showed the highest values in Tier-1 swimmers (Table 4). For instance, this was the case of *D_a_* or *Re* that were significantly higher in top-tier swimmers. Conversely, parameters that are also anthropometrics-dependent, such as *Fr* and *v_h_* Top-tier swimmers exhibited the best scores. There was no significant variation across groups in the *C_Da_*.

**Table 4:** Comparison of the drag and dimensionless hydrodynamic variables.

	Descriptive			ANOVA			*Post hoc*		
	Tier-1	Tier-2	Tier-3	F-ratio	P	$\eta_{p}^{2}$	T1vT2	T1vT3	T2vT3
	Mean ± 1SD	Mean ± 1SD	Mean ± 1SD	(2;72)			p	p	p
	(95CI)	(95CI)	(95CI)				(d)	(d)	(d)
*D_a_*	74.22 ± 33.38	45.18 ± 14.16	44.02 ± 27.42	10.621	<0.001	0.23	<0.001	<0.001	1.00
[N]	(63.75;84.69)	(34.72;55.65)	(33.56;54.49)				(1.13)	(0.99)	(0.05)
*C_Da_*	0.41 ± 0.17	0.35 ± 0.15	0.37 ± 0.17	0.615	0.54	0.02	0.89	1.00	1.00
[dimensionless]	(0.34;0.47)	(0.28;0.42)	(0.30;0.43)				(0.37)	(0.24)	(0.13)
*Fr*	0.37 ± 0.02	0.34 ± 0.01	0.30 ± 0.02	96.730	<0.001	0.73	<0.001	<0.001	<0.001
[dimensionless]	(0.36;0.38)	(0.33;0.35)	(0.29;0.31)				(1.90)	(3.50)	(2.53)
*v_h_*	1.62 ± 0.04	1.60 ± 0.03	1.58 ± 0.04	7.893	<0.001	0.18	0.04	<0.001	0.47
[m/s]	(1.61;1.64)	(1.58;1.61)	(1.57;1,60)				(0.57)	(1.00)	(0.57)
*Re*	2.82 ± 2.05	2.44 ± 1.08	2.11 ± 1.93	103.168	<0.001	0.74	<0.001	<0.001	<0.001
(× 10^6^) ± (× 10^5^)	(2.75;2.88)	(2.37;2.50)	(2.04;2.18)				(0.23)	(0.37)	(0.21)


### Mechanical Power

Regarding mechanical power, Tier-1 swimmer showed significantly higher values than other two groups in all selected parameters (Table 5). There was no significant differences between the two other groups. Tier-1 swimmers delivered more power to overcome drag, transferring kinetic energy to water, and therefore more external power and power input.

**Table 5:** Comparison of the mechanical power and energy expenditure.

	Descriptive			ANOVA			*Post hoc*		
	Tier-1	Tier-2	Tier-3	F-ratio	P	$\eta_{p}^{2}$	T1vT2	T1vT3	T2vT3
	Mean ± 1SD	Mean ± 1SD	Mean ± 1SD	(2;72)			p	p	p
	(95CI)	(95CI)	(95CI)				(d)	(d)	(d)
*ẇ_d_*	113 ± 54	61 ± 19	53 ± 35	17.827	<0.001	0.33	<0.001	<0.001	1.00
[W]	(97;128)	(46;77)	(38;69)				(1.29)	(1.32)	(0.28)
*ẇ_k_*	303 ± 146	182 ± 86	182 ± 108	9.016	<0.001	0.20	<0.001	<0.001	1.00
[W]	(257;349)	(135;228)	(136;229)				(1.01)	(0.94)	(0.00)
*ẇ_ext_*	416 ± 186	243 ± 98	235 ± 138	2.339	<0.001	0.26	<0.001	<0.001	1.00
[W]	(358;474)	(185;301)	(177;294)				(1.16)	(1.11)	(0.07)
*Ė_tot_*	2080 ± 929	1216 ± 489	1177 ± 692	12.339	<0.001	0.26	<0.001	<0.001	1.00
[W]	(1696;2463)	(1014;1417)	(891;1462)				(1.16)	(1.10)	(0.07)


## Discussion

The aim was to compare the anthropometrics, biomechanics and energetics in age-group swimmers of different competitive levels. It was noted significant variations, with moderate-large effect sizes, among the three tiers, for most variables selected in this research. Performances by Tier-1 swimmers were related to their anthropometrics, biomechanics and energetics.

### Anthropometrics

Literature is consistent reporting that the best age-group swimmers are prone to be taller, heavier and having long limbs (Morais et al., 2012; Barbosa et al., 2015a). For instance, after a summer break of 10-weeks, swimmers grew-up, lengthen the arm span, had larger hands and feet, increased *SL, SI*, η_F_, and delivered better performance than before (Moreira et al., 2014). When 94 young swimmers were followed-up over 3 years, arm span (as a proxy of anthropometrics) was retained in the output of a hierarchical linear model predicting swimming performance (Morais et al., 2017). Hence, regardless of the training or detraining program young swimmers are under, they are prone to improve their performances due to growth and maturation. The rate of growth and maturation is genetically determined, having each child different rates and, spurs happening in different moments of their development. Therefore, swimming fraternity involved in age-group swim should be aware of this phenomenon as there is very high between-subjects variability, leading to a significant amount of shifts in tier membership over time (Morais et al., 2015).

### Stroke Kinematics

Being swimming a periodic motion, average velocity depends on the *SL* and *SF* (v = SL SF). As happened in Tier-1 swimmers, a long *SL* concurrent to a fast *SF* elicits a faster speed. Conversely, short *SL* and slow *SF* leads to slow speed as happened in Tier-3 swimmers. Tier-2 had long *SL* but slow *SF*. Nevertheless, different *SL-SF* combinations enable one to reach a given speed. Therefore, the question to be raised is if, at a given velocity, the best option is to increase the *SF* or *SL*. Analytical models have proposed a relationship between swim efficiency and *SL* (Toussaint and Hollander, 1994):

(17) $$SL=\sqrt[{3}]{\frac{{\text{η}}_{\text{p}\cdot w}}{D\cdot SF^{2}}}$$

Where *SL* is the stroke length, η_p_ the propelling efficiency, *w* the mechanical work per stroke cycle, *D* the drag force and *SF* the stroke frequency. A longer *SL* would be related to better efficiency and higher mechanical work per stroke. Experimental studies recruiting adult swimmers have failed to confirm clearly this argument (Pendergast et al., 2003; Zamparo et al., 2005; Barbosa et al., 2008). Nonetheless, research on age-group swimmers has pointed out for the importance of a long *SL*. Structure equation modeling retained *SL* as a factor determining *SI* (Morais et al., 2012). Multifactorial models pointed out for same trend of *SL* being a strong predictor of performance (Saavedra et al., 2010). Surprisingly, following-up over 3 years young swimmers, *SL* was a strong negative predictor of performance when analyzed by hierarchical linear modeling (Morais et al., 2017). The explanation furnished in this latter research was that swimmers could be transitioning from prepubertal to peripubertal maturational stages at some point, affecting their motor control patterns. Coordination patters emerge from the continuous interaction of the constraints acting on the individual. So, there is the need of individual adaptations to interacting constraints (environmental, organismic and task constraints) (Seifert et al., 2014). Constraints acting on performance and training behaviors are more often temporary than permanent, and their influence can be strengthened or reduced according to different time scales (Harbourne and Stergiou, 2009). As such, Tier-2 swimmers are constraining their *SL-SF* combination with the understanding that increasing *SL* it will make them reach a faster speed as efficiently as possible.

### Swim Efficiency

Tier-3 swimmers were less efficient than the other two tiers. There was no difference between Tier-1 and Tier-2. Again, structural equation modeling has already proposed that swim efficiency is a performance-determinant in age-group swimming (Barbosa et al., 2010; Morais et al., 2012). Average and range values of *dv* (Barbosa et al., 2013), η_F_ (Zamparo, 2006; Morais et al., 2017) and *SI* (de Mello Vitor and Böhme, 2010; Morais et al., 2015) are within what is reported in the literature for this age bracket. A high *dv* is reported as related to poor time trials and more energy cost of swimming (Barbosa et al., 2010). Swimming, acceleration is the net balance between thrust and resistance. Deriving the Newton’s second law:

(18) $$a=\frac{Pr-D}{BM+m_{a}}$$

Where *a* is the swimmer’s acceleration, *Pr* the thrust, *D* the drag force, *BM* the swimmer’s body mass and *m_a_* the added water mass. Such accelerations over the cycle, lead to:

(19) $$v=v_{0}+\Delta v(t)$$

Where *v* is the swimmer’s mean velocity, *v_0_* the swimmer’s velocity at the beginning of the stroke cycle, Δv the variation of the swimming velocity throughout the stroke cycle and *t* the time. Hence, swimmers are not able to keep an uniform motion (i.e., Δ*v* = 0 m/s). Instead, they denote an intra-cyclic variation of the velocity (i.e., Δ*v* ≠ 0 m/s). In the event of a higher dv (i.e., Δ*v* in equation 19) for same average velocity, more work is needed to overcome inertia and drag:

(20) $$w=K\cdot v_{0}^{3}\cdot T$$

(21) $$w=K\cdot {\left(v_{0}+|\Delta v|\right)}^{3}\cdot T$$

Where *w is the* mechanical work, *K* drag factor, *v* swimming velocity and *T* duration of a stroke cycle.

η_p_ is the ratio of total work or power to overcome drag force and displace in water, whereas η_F_ is the ratio of external work or power to overcome this same force. Indeed, we did not account for *ẇ_int_*. There are a few challenges to measure *ẇ_int_* in water. A main concern is the shift of the center of mass over the stroke cycle which is deemed to potentially violating König’s theorem. By König’s second theorem, the kinetic energy is the sum of the kinetic energy of the center of mass and kinetic energy of moving parts of the body having as reference the center of mass (Rao, 2005):

(22) $${}_{}^{N}E_{k}=\frac{1}{2}\cdot m\cdot_{}^{N}v\cdot_{}^{N}v+\frac{1}{2}\cdot_{}^{N}H\cdot_{}^{N}{\text{ ω}}^{R}$$

Where, *Ek* is the energetic kinetic, *N* the reference frame, *m* the mass, *v* velocity of the center of mass, *H* angular momentum, *ω* angular velocity of rigid body *R*. Total kinetic energy must be greater or equal to kinetic energy of the center of mass. In swimming, the center of mass denotes a quite large volume of displacement leading to substantial inaccuracies measuring the *ẇ_int_*.

An interesting note is that η_F_ ranged overall 21–30% for pooled sample. Humans walking on soft and slippery surface (i.e., sand) were reported as having a gait efficiency of 30–60% between 0.5 and 2.5 m/s (Lejeune et al., 1998). In this same study, walking at about 1.25 and 1.50 m/s the efficiency was approximately 44 and 50%, respectively. Adult competitive swimmers have a η_F_ of 50–60% (Pendergast et al., 2003). I.e. skilful young humans displacing in water can be almost as efficient as adults walking on soft sand. Overall, swim efficiency can be deemed as an important factor to reach better performances in age-group swimmers.

### Hydrodynamics

Hydrodynamics is arguably the domain where is less easy to find a clear trend. Parameters that are speed-dependent, such as *D_a_*, turned out to be a disadvantage to better swimmers. Re-arranging equation 9, becomes clear the key-role played by the velocity term in the magnitude of the drag force:

(23) $$D_{a}=\frac{1}{2}\cdot \text{ ρ}\cdot v^{2}\cdot S\cdot C_{Da}$$

Active drag depends on the square of the velocity. Tier-1 swimmers because swam faster the time trials, the drag to be overcome was likewise larger than their counterparts. Conversely, there was no significant variation across groups in the *C_Da_*. Benchmarking with literature, *C_Da_* of these participants is smaller. For pooled sample, the 95CI was 0.30–0.47. Over 2.5 years, the *C_Da_* assessed by MAD system, decreased from a 95CI of 0.60–0.68 to 0.49–0.59 (Toussaint et al., 1990). In a research that young swimmers were monitor in three different moments of a season by VPM, the 95CI brackets were 0.41–0.55, 0.55–0.97, and 0.33–0.47 (Barbosa et al., 2015b). The 95CI of present research seems to overlap the data reported by the end of the season in the latter paper.

Typically, it is deemed a turbulent flow when *Re* > 5 × 10^5^. The lower limit of the 95CI in Tier-3 was 2.04 × 10^6^. This suggests that regardless of the tier, young swimmers are under turbulent conditions. *Re* was different across all tiers and higher in Tier-1. The difference in *Re* across tiers is explained by swim velocity each tier is able to reach and also the anthropometrics as discussed previously.

Other parameters that are anthropometric-depended, such as *Fr* and *v_h_*, Tier-1 swimmers showed better scores. Both variables provide insight on wave-making resistance. Literature reports that swimmers with longer bodies (i.e., taller) benefit in decreasing the wave drag (Kjendlie and Stallman, 2008). This reasoning comes from naval engineering where ships with long hulls are under less wave drag. The 95CI of the *v_h_* in our three tier was 1.61–1.64, 1.58–1.61, and 1.57–1.60 m/s. Their 95CI of the swim velocity was 1.48–1.53, 1.33–1.38 and 1.17–1.22 m/s. Hence, swim velocity falls within the *v_h_*, suggesting that swimmers were displacing within wake wavelength. Moreover, there is room to increase the swim velocity by about 0.11–0.12, 0.23–0.25, and 0.38–0.40 m/s with negligible change in the wave drag.

### Mechanical Power

Tier-1 swimmers delivered more mechanical power. In another research recruiting swimmers of the same age-group, percentile 10–90 for *ẇ_D_, ẇ_k_*, and *ẇ_ext_* was 38–137, 96–282 and 135–329 W, respectively (Barbosa et al., 2015a). At 1.25 m/s the *ẇ_D_* on MAD system was noted increasing from 52 ± 11.58 W to 77.2 ± 14.81W over a 2.5 years period in 13 young swimmers (Toussaint et al., 1990). In another study, at 1.2–1.4 m/s *ẇ_D_, ẇ_k_* and *ẇ_ext_* were 74–97, 81–112 and 179–245, respectively (Zamparo et al., 2005). These values fall within the 95CI of our results. Tier-1 swimmers, as adult counterparts, use a much higher proportion of their *ẇ_ext_* to overcome drag (Toussaint, 1990). This can be the result of a more efficient swim technique as discussed early on and, highest levels of dry-land strength and power being transferred into the swim stroke (Morais et al., 2018). High levels of dry-land strength that are transferred to in-water thrust elicit the swimmer to reach faster speeds (Morouço et al., 2011).

The power input was also higher in Tier-1 swimmers. This can be explained by their larger surface areas and speeds, as well as, having to overcome a higher drag force in comparison to counterparts. The mean values of each group are within what is reported in the literature selecting experimental techniques. For instance, the mean *Ė_tot_* was for Tier-1, -2, and -3 2080 ± 929 W at 1.51 ± 0.06 m/s, 1216 ± 489 W at 1.35 ± 0.04 m/s, and 1177 ± 692 W at 1.19 ± 0.08 m/s, respectively. In Tier-1 was noted a large SD, that is almost the double of remaining groups. In Tier-1 four swimmers seem to be extreme cases (*Ė_tot_* > 3 kW) due to large trunk transverse surface areas. Removing these 4 extreme cases, the mean *Ė_tot_* would be 1742 ± 484 W. Zamparo et al. (2005) reported *Ė_tot_* of 857 W at 1.2 m/s and 1126 W at 1.4 m/s. Pendergast et al. (2003) proposed that the *Ė_tot_* –speed at 1.1–1.3 m/s should be around 1.0–1.4 kW. In a 200 m time trial, at 1.42 ± 0.05 m/s the *Ė_tot_* was 2.23 ± 0.23 kW (Figueiredo et al., 2011). Hence, the *Ė_tot_* derived in this research seems to be a fair approximation of what is reported by experimental studies. This analytical procedure can have good traction in settings where experimental testing is not feasible. For instance, events at official competitions, assessing large sample sizes, and facing time-constrains to monitor swimmers, equipment available does not fit the participant’s size (e.g., equipment is too large for small children). That said, to minimize the source of variability, it is advised to select always the same procedure and input the same assumptions running within- and between-subjects comparisons.

### Benchmarking Strongest and Weakest Domains

Benchmarking the strongest and weakest domains of each tier it is possible to have insight on the determinant factors explaining their performance (Table 6). Anthropometrics (to be taller, having longer limbs and heavier) is an advantage for Tier-1 swimmers. Anthropometric features are quite similar between Tier-2 and Tier-3. Tier-1 had long *SL* and high *SF* leading to a fast speed. Tier-3 on the other hand had short *SL* and slow *SF*. Conversely, Tier-2 was in the middle ground, having high *SL* but slow *SF*. As far as swim efficiency is concerned, Tier-3 swimmers were less efficient than the other two tiers. Hydrodynamics is arguably the domain where is less easy to find a clear trend. There was no significant variation across groups in the *C_Da_*. But, parameters that are speed-dependent turned out to be a disadvantage for better swimmers, whereas, parameters that are anthropometric-depended they showed better scores. Tier-1 swimmers yielded larger external power and power input, which might be related to faster speeds, anthropometric features and swim technique. No differences were noted between the other two groups under comparison. As such, adding to the nature-nurture debate, one can argue that both are useful to excel at such early ages. The combination of nature (having here the anthropometrics as proxy) and nurture (swimming biomechanics and energetics) is the optimal solution. Mid-tier swimmers seems to be those that despite the anthropometrics is not yet on their favor, nurture has help them in some way to reach good performances. Low-tier swimmers are those lacking both “nature” and “nurture” features.

**Table 6:** Benchmark of the strongest and weakest domains of each tier.

	Anthropometrics	Kinematics	Efficiency	Hydrodynamics	Power
Tier-1	++	++	++	=	++
Tier-2	=	+	+	=	=
Tier-3	=	-	-	+	=

	**Nature**	**Nurture**		

Tier-1	+	+		
Tier-2	-	+		
Tier-3	-	-		


*++ likely a large advantage to remaining tiers, + likely a moderate advantage, - likely a moderate disadvantage, = likely a trivial advantage between two different tiers.*

It can be addressed as limitations of this research: (i) this is a cross-sectional research. As such, data may vary along the season depending on training regime and individual rates of growth and development; (ii) one should exercise care extrapolating these findings to other cohorts of swimmers. Findings can vary in other age-groups and in girls; (iii) mechanical power output and power input were derived from a set of analytical formulae albeit, experimental procedures are also a potential alternative; (iv) all measurements were took considering a non-competitive distance. The shortest competitive distances in swimming are the 50 m and 50 y (approximately 45 m) events. 25 m trials were selected because it is very convenient. In a short and quick trial, it is possible to collect a comprehensive number of valid parameters. Moreover, there was a strong association (*R* = 0.79) in the speed over the 25 m bout and the 100 m race speed by our participants.

## Conclusion

As a conclusion, it was noted significant variations, with moderate-large effect sizes, among the three tiers, for the vast majority of the selected variables. The better performances by Tier-1 swimmers were related to their anthropometrics, biomechanics and energetics.

## Ethics Statement

This study was carried out in accordance with the recommendations of Declaration of Helsinki. All subjects gave written informed consent. The protocol was approved by the Ethic Committee of the University of Trás-os-Montes and Alto Douro.

## Author Contributions

TB, MC, and JM conceived and designed the experiments. RB and JM performed the experiments. RB, JM, TB, and MC analyzed the data and drafted the manuscript.

## Conflict of Interest Statement

The authors declare that the research was conducted in the absence of any commercial or financial relationships that could be construed as a potential conflict of interest.
